# PALS1-dependent modulations of mRNA profiles in MDCK II cells grown in non-confluent monolayers and three-dimensional cysts

**DOI:** 10.1186/s12863-024-01284-0

**Published:** 2024-11-29

**Authors:** Klaus Schughart, Annika Möller-Kerutt, Verena Höffken, Pavel Nedvetsky, Ann-Christin Groh, Daniela Anne Braun, Hermann Pavenstädt, Thomas Weide

**Affiliations:** 1 Institute of Virology Münster, University of Münster, 48149 Münster, Germany; 2 Department of Microbiology, Immunology and Biochemistry, University of Tennessee Health Science Center, Memphis, TN 38103 USA; 3 Department Molecular Nephrology, Internal Medicine D (MedD), University Hospital of Münster (UKM), 48149 Münster, Germany; 4 Department Medical Cell Biology, Internal Medicine D (MedD), University Hospital of Münster (UKM), 48149 Münster, Germany

**Keywords:** *PALS1*, Crumbs complex, Cell polarity, Junctions, Cell contacts, RNA sequencing, MDCK II, GO term enrichment studies

## Abstract

In epithelia, apicobasal cell polarization is closely linked to cell-cell contact formation, both controlled by the conserved Crumbs (CRB) complex, which includes the transmembrane protein Crumbs (CRB3a) and adapter proteins PALS1, PATJ, and LIN7c. In MDCK II cells, a model for cell polarization, depletion of PALS1 - which binds to all CRB components - leads to defective cell polarization and improper distribution of tight junction proteins, resulting in severe epithelial barrier defects in 3D cyst models. This study investigated whether this phenotype is associated with transcriptional changes by analyzing wildtype (WT) and *PALS1* knockout (KO) MDCK II cell lines grown under non-confluent conditions and in 3D cyst cultures. Our results indicate that the transition from non-confluent cells to 3D cysts involves numerous differentially expressed genes (DEGs) in both WT and KO cells. Importantly, the analyses revealed significant overlaps between WT and KO cells in their maturation processes, suggesting that most identified DEGs are linked to differentiation from non-confluent to polarized MDCK cells and likely not a result of PALS1 deficiency. Gene Ontology (GO) enrichment and over-representation analyses using REACTOME and KEGG databases confirmed these similarities. In contrast, the direct comparison of WT and KO cells at the two stages showed fewer DEGs and overlaps in associated biological processes and signaling pathways. DEGs associated with the 3D stage, in which the phenotype manifests, contain DEGs and pathways that were predominantly linked to cell cycle linked processes, centromere assembly, or DNA replication. Furthermore, the transcription of genes encoding key junction proteins, additional polarity proteins, and cell-substrate interaction proteins is less affected by the loss of PALS1, indicating that PALS1 influences the transcriptional profiles in epithelial cells as a modulating factor.

**Supplementary Information:**

The online version contains supplementary material available at 10.1186/s12863-024-01284-0.

## Background

The CRB complex plays a key role for cell polarization and tight junction (TJ) formation. It is formed by members of the CRB protein family (CRB1, CRB2 and the two CRB3 splice isoforms CRB3a and CRB3b), LIN7c (lin-7 homolog C), and PALS1 (protein associated with LIN7c) and PATJ (PALS1-associated tight junction protein). Members of the CRB protein family are type I membrane proteins, passing the plasma membrane once, whereas the other components of the CRB complex are cytoplasmic scaffold proteins [1, 2]. Of note, solely PALS1 interacts with all components of the Crumbs core complex. Via two L27 domains (L27N and L27C) PALS1 binds to LIN7c and PATJ, respectively [3–5]. The C-terminal part of PALS1 contains a PDZ, an SH3, and a GUK domain, also called PSG module, and mediates the interaction to the cytoplasmic tails of all CRB proteins, except for the splice variant CRB3b [5–7]. The N-termini of the two PALS1 isoforms contain an evolutionarily conserved region (the ECR) that carries a binding site for the protein PAR6 [8, 9]. PAR6 in turn is part of a second crucial polarity complex, called PAR complex, which is composed of the adaptor proteins PAR3 and PAR6 and the atypical protein kinase C (aPKC) [2]. Experiments in epithelial cell culture models elucidated that knockdown or knockout of PALS1 result in defective apicobasal polarization and cell junction formation [10–12].

In vivo, a lack of PALS1 in nephrons leads to a lethal phenotype, accompanied by an increased formation of renal cysts [13, 14]. This phenotype indicates a pivotal role of PALS1 for the establishment of functional nephron segments in mammalian kidneys and resembles cysts caused by the lack of the PALS1 binding partners LIN7c and CRB3a, or of TAZ, a central co-transcriptional activator of the Hippo signaling [15–21]. Remarkably, the loss of only one *PALS1* allele is sufficient to cause this renal phenotype, arguing for gene-dose-dependent functions of PALS1 in renal epithelial cells [13]. To address whether reduced PALS1 expression levels affect cell polarization, or rather cause the defects in the formation of cell contacts, we recently established Madin Darby canine kidney (MDCK II) cells lacking *PALS1* [12]. The results demonstrated a delayed formation of TJ accompanied by the strongly reduced lateral distribution of TJ proteins along bicellular junctions and confirmed in vitro studies done in mammalian cell culture systems using other components of the CRB complex (e.g. PATJ) [12, 22–24]. The misdistribution of TJ protein (e.g. ZO-1 and Occludin) in *PALS1* knockout cells causes increased paracellular permeability and severe epithelial barrier defect as well as a strongly increased formation of multiple lumens in three-dimensional spherical cysts (3D cysts), indicating malformed apicobasal cell polarization [10, 12].

The aim of this study is to analyze how this phenotype is connected to changes at the transcriptional level. To do this, we examined the transcriptomes of wild-type (WT) and PALS1 knockout (KO) cells grown either under non-confluent (nc) conditions or in 3D cysts (3D). The analyses focused on which genes are differentially expressed between unpolarized and polarized cells, as well as between WT and KO cells in these stages. The results suggest that PALS1 may primarily play a role in fine-tuning the balance between epithelial differentiation and cell cycle progression.

## Methods

### Generation and establishment of PALS1 knockout cell lines

MDCK II (thereafter MDCK) cells were generated as described earlier [12]. In brief, to generate a PALS1 knockout in MDCKII cells (Sigma, #ECACC 00062107) frameshift mutations were introduced via CRISPR/Cas9. CRISPOR was used to design the following guide RNA/DNA-sequences CACCGATCATTAGTCGGATAGTAAA, AAACTTTACTATCCGACTAATGATC. The gRNAs/gDNAs (100µM, 8 µl each) were phosphorylated and annealed via adding 2 µl 10x PNK buffer, 1 µl ATP (5mM) and 1 µl Polynucleotide Kinase and incubation for 1 h at 37 °C followed by incubation at 95 °C for 5 min to stop the reaction. The resulting oligonucleotides were inserted into the px459 plasmid via digestion using BbsI and ligation by T4 Ligase. MDCKII cells with a confluency of 60% were transfected using Lipofectamine™ 2000 following the manufacturer’s protocol. Selection was done using puromycin (4 µg/ml) for 48 h. Potential knockout cells were separated to analyze single cell clones. Potential clones were sequenced and only clones with frameshift mutations were kept.

### Cultivation of MDCK II cell lines

MDCK cell lines were grown as described earlier [12]. Cells were grown in modified Eagle’s medium (MEM, Lonza) containing 5% fetal calf serum (FCS), 2 mM L-Glutamine and 1% 100x Penicillin/Streptomycin (P/S). The cells were cultivated at 37 °C and 5% CO_2_. To form cysts MDCK cells were seeded in a matrix hydrogel (Cultrex PathClear Reduced Growth Factor BME, Type 2, from R&D Systems) for seven days, before using them for further analyses.

### RNA isolation and RNA sequencing

RNA isolation from WT and PALS1 KO cells grown non-confluent and in 3D cysts was performed using the GenElute Mammalian Total RNA Miniprep Kit (Sigma Aldrich, #RTN350) following the manufacturer’s instructions. Briefly, cells were lysed and homogenized, RNA was cleared via filter columns and washed with buffer and ethanol before eluted in 50 µl elution buffer. Isolated RNA was stored at -80 °C until further proceeding.

Sequencing was performed at the Core Facility Genomics of the Medical Faculty Münster. Quality and integrity of total RNA was controlled on Agilent Technologies 2100 Bioanalyzer (Agilent Technologies; Waldbronn, Germany). PolyA^+^RNA was purified from 100 ng total RNA using Poly(A) mRNA Magnetic Isolation module Kit (NEB E7490L, New England Biolabs). The RNA Sequencing library was prepared with NEB Next^®^ Ultra™ II Directional RNA Library Prep Kit for Illumina^®^ (New England Biolabs). The libraries were sequenced on Illumina Nextseq 2000 using NextSeq2000 P3 Reagent Kit (88 cycles, single reads run 72 bp) with an average of 26.9 million reads per RNA sample.

### Bioinformatic analyses

Reads were quality checked with package FastQC (version 0.11.9, http://www.bioinformatics.babraham.ac.uk/projects/fastqc), then trimmed using Trimgalore version 0.6.7 (https://www.bioinformatics.babraham.ac.uk/projects/trim_galore/) with default settings. Trimmed reads were mapped to the dog genome (*Canis_lupus_familiaris*, ENSEMBL, release 108) using STAR version 2.5.2b [25]) with default settings. Mapped reads were counted at the gene level using RsubRead version 1.32.4 [26]. Analysis and visualization of expression data was then performed using the R software package version 4.2.1 [27], (http://www.R-project.org) and RStudio version 2022.07.2 (https://www.rstudio.com). Raw counts were normalized and log_2_ transformed using function rlog Transformation from the DESeq2 package version 2.7.9a [28], and an increment was added to the normalized values to make all values positive. For identification of differentially expressed genes (DEGs), DEseq2 was used with the model design = ~ group and subsequently contrasting groups: group 1: WT-nc (Ctr_2D_nconf), group2: WT-3D (Ctr_3D_7d ), group 3: KO-nc (KO_2D_nconf), group 4: KO-3D (KO_3D_7d) with a threshold for the adjusted p-value of < 0.05 and more than a 2-fold (|log2|> 1) difference for expression levels. Volcano plots were generated with the package Enhanced Volcano, version 1.14.0 (https://github.com/kevinblighe/EnhancedVolcano) [29]. Functional analyses of DEGs were performed using the R software package EnrichR version 3.1 [30]. For beeswarm graphs of expression levels, package beeswarm version 0.4.0 [31] was used (https://CRAN.R-project.org/package=beeswarm). Heatmaps were generated with the function heatmap2 of package gplots version 3.1.3 (https://github.com/talgalili/gplots) and package pheatmap [32]. Venny 2.1.0 was used for generation of intersections (https://bioinfogp.cnb.csic.es/tools/venny/index.html) [33] and (Corel Draw X6 was used for final mounting of figures).

### Data Availability

The raw sequences will be available at the GEO public database, ID: GSE264311 (GEO expression database. https://www.ncbi.nlm.nih.gov/geo).

## Results

### Polarization of WT and PALS1-depleted MDCK cells results in strong changes of their mRNA expression profiles

Non-confluent cells have only partial formed cell-cell contacts and are less polarized. In contrast, MDCK cells, that were embedded in extracellular matrix (ECM), form 3D cysts with a single apical lumen. Hence, cysts are formed by highly polarized epithelial cells with a defined separation into apical and basolateral membrane domains, separated by tight and adherence junctions and share many properties with in vivo epithelia [34]. Thus, non-confluent cells and well-polarized cells lining the 3D cyst represent two extreme states of an epithelial cell with obvious and significant changes in cell morphology and shape: on the one hand, an immature, proliferating and marginally polarized state, and on the other hand a mature, highly polarized state with rather low levels of proliferation.

Here, we analyzed transcriptomes of non-confluent (nc) cells and of cells grown in 3D cysts (3D) from the starting wild-type cell line, cells that ran through the CRISPR/Cas9 process but without a targeting RNA for the *PALS1* gene (control cells), and MDCK knockout cells with a defective *PALS1* gene (KO cells) [12]. Since the transcriptomes of the starting wild-type cell pool and non-targeted CRISPR/Cas9 control cells grouped close together in a principal component analysis, we combined the non-confluent samples into the ‘WT-nc’, and 3D-cyst samples into the ‘WT-3D’ group (Fig. SF1; Tab. ST1).

**Fig. 1:**
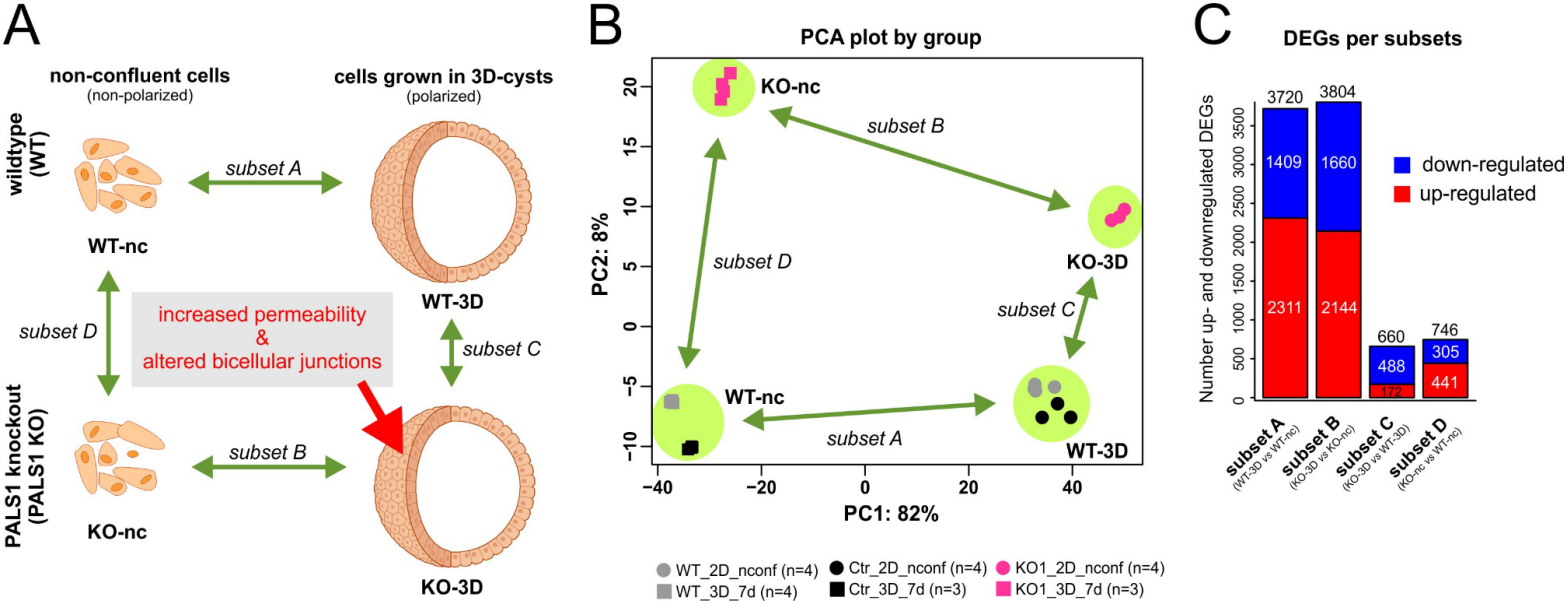
Analyses of the mRNA profiles of wildtype and *PALS1* KO MDCK cells grown under non-confluent conditions and in 3-dimensional cysts. **A**: Schematic overview of the experimental setup and the four performed comparisons. The transcriptomes of non-confluent MDCK wildtype cells were compared to cells grown in 3D cyst cultures (WT-3D vs. WT-nc). The differentially expressed genes (DEGs) of this comparison were called *subset A*. The analogous approach done with *PALS1* knockout (KO) MDCK cells (KO-3D vs. KO-nc) represents the *subset B* of DEGs. The comparison of transcriptomes of WT and KO 3D cysts (KO-3D vs. WT-3D) resulted in DEGs that are represented by *subset C*. The analogous comparison of non-confluent WT and KO cells is subset D (KO-nc vs. WT-nc). **B**: PC1 and PC2 of principal component analysis normalized transcriptome expression values from all replicates of experimental groups. Samples of the starting wildtype cell pool, and a control cell line in which the *PALS1* gene remained unaffected after the CRISPR/Cas9 mutagenesis process were combined leading to four different groups (WT-nc, KO-nc, WT-3D, and KO-3D, Fig. SF1). Only minor differences were apparent between CRISPR/Cas9 controls and untreated WT cells, both at non-confluent and at 3D cyst stages. Each dot represents an independent biological replicate. A clear separation was apparent between WT and KO and non-confluent and 3D cyst groups. Replicates grouped well together. **C**: Number of total-, up- and downregulated DEGs of *subset A and subset B*, and *subset C and subset D.* Subsets A and B showed a much higher number of DEGs in comparison to subset C and D

This resulted in four groups of mRNA profile samples (Fig. 1A): first, transcriptomes from non-confluent WT cells (group 1: WT-nc), second, transcriptome samples of WT cells grown in 3D cysts (group 2: WT-3D), third, transcriptomes of MDCK *PALS1* KO cells grown under non-confluent conditions (group 3: KO-nc), and fourth, *PALS1* KO cells grown in 3D cysts (group 4: KO-3D). A principal component analysis (PCA) of the normalized expression levels revealed a clear separation of these four groups (Fig. 1B).

Next, we compared the expression profiles from WT cells grown in 3D cysts to non-confluent WT cells (WT-3D *versus* WT-nc; subset A; Tab. ST2A). The same comparison was made with the mRNA profiles of KO cells (subset B, Tab. ST2B). These comparisons revealed 3720 DEGs for WT cells, of which 2311 were up- and 1409 downregulated in 3D cysts (subset A). The KO differentiation process into 3D cysts (subset B) was linked to 3804 DEGs, of which 2144 were up - and 1660 downregulated in 3D cysts.

Subset C represents the direct comparison of the mRNA profiles of 3D cysts from KO versus WT cells (subset C, Tab. ST2C) and subset D the corresponding comparison of non-confluent KO and WT cells (KO-nc *versus* WT-nc, subset D). The comparison of WT and KO cells grown in 3D cysts identified 660 DEGs (subset C, Tab. ST2C), of which 172 DEGs were up- and 488 downregulated. Finally, the comparison of non-confluent WT and KO cells resulted in 746 DEGs, including 441 upregulated and 305 downregulated genes (subset D, Tab. ST2D, Fig. 1C). The DEGs expression levels of subset C and D were normalized to the values of KO cells.

**Fig. 2:**
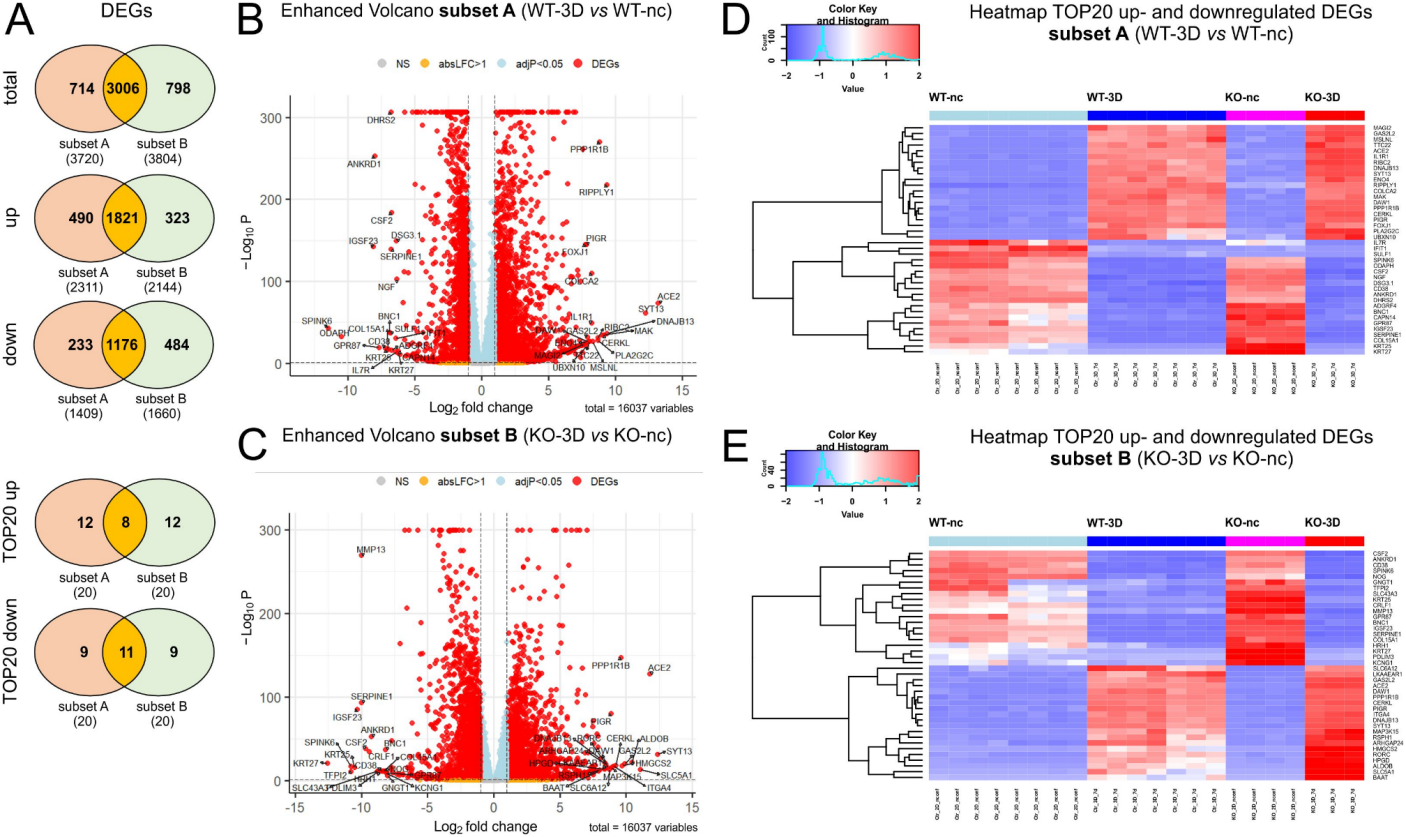
Summary of DEGs of WT and *PALS1* KO MDCK during cell polarization. **A**: Venn diagrams illustrating overlaps of total (upper diagram), upregulated (middle diagram) and downregulated DEGs of subsets A and subset B. The two down Venn diagrams show shared genes of the top 20 up- or downregulated DEGs of the subsets A and B. In all cases most of the DEGs were shared by subsets A and B. B and C: Volcano plots of DEGs of *subsets A* (**B**) and *subset B* (**C**), with annotated TOP20 up- and downregulated genes. y-axis: -log_10_ adj. P, x-axis: log_2_ fold change. DEGs are colored red and the TOP20 up- and down-regulated (by log-fold change) DEGs are labeled. Blue: genes with adj. *P* < 0.05. Yellow: genes with absolute log_2_-fold change > 1. D and E: Heatmap of TOP20 upregulated (**D**) and downregulated DEGs (**E**) showing the difference of relative gene expression levels in WT and *PALS1* KO MDCK cells

To elucidate similarities and differences between the WT and KO cells we next applied intersection analyses. Most of the DEGs linked to the WT differentiation (subset A) were also differentially expressed in KO cells (subset B; 66.5% equivalent to 3006 DEGs). Only 15.8% (714 DEGs) and 17,7% (798 DEGs) were exclusively regulated in WT or KO cells, respectively. A similar pattern was observed if only up- or downregulated DEGs of subsets A and B were considered separately (Fig. 2A, Tab. ST3). The Volcano plots illustrate the distribution of the DEGs analyses for the WT (Fig. 2B) and KO DEGs (Fig. 2C). The twenty strongest (by log-fold change) upregulated (TOP20-up) and downregulated (TOP20-down) genes that are shown in the heatmaps in Fig. 2D, E.

These analyses showed that eight of the TOP20 upregulated DEGs *(ACE2*, *SYT13*, *DNAJB13*, *CERKL*, *PPP1R1B*, *DAW1*, *GAS2L2*, *PIGR)* were shared by subsets A and B. Twelve DEGs were either only found in subset A *(RIPPLY1*, *MAK*, *PLA2G2C*, *RIBC2*, *MSLNL*, *COLCA2*, *L1R1*, *TTC22*, *UBXN10*, *ENO4*, *FOXJ1*, *MAGI2)* or in the subset B *(SLC5A1*, *ALDOB*, *HMGCS2*, *ITGA4*, *MAP3K15*, *SLC6A12*, *ARHGAP24*, *LKAAEAR1*, *RORC*, *HPGD*, *RSPH1*, *BAAT)*(Tab. ST3).

In the case of the TOP20 downregulated DEGs, eleven DEGs were common in subsets A and B *(SPINK6*, *IGSF23*, *ANKRD1*, *GPR87*, *CD38*, *KRT25*, *COL15A1*, *BNC1*, *SERPINE1*, *CSF2*, *KRT27)*. Nine genes were only found in subset A *(ODAPH*, *SULF1*, *DHRS2*, *ADGRF4*, *IFIT1*, *IL7R*, *DSG3.1*, *NGF*, *CAPN14)* or subset B *(TFPI2*, *MMP13*, *CRLF1*, *PDLIM3*, *SLC43A3*, *HRH1*, *NOG*, *KCNG1*, *GNGT1)* (Tab. ST3). Of note, the majority of the top 40 regulated DEGs of subset A (38 DEGs out of the TOP20 up and TOP20 down DEGs) were also significantly regulated DEGs in KO cells. Only the genes *IFIT1* and *SULF1* were exclusively found in subset A. In the case of the top regulated 40 DEGs of the KO cells, all these DEGs were found to be significantly regulated in WT cells (Tab. ST3).

Together, the analyses showed strong overlaps between subsets A and B. This indicates that the majority of the identified DEGs of subset A and B are linked to the differentiation process from non-confluent to polarized MDCK cells and are most probably not related to *PALS1*-deficiency. However, a smaller group of DEGs were pronounced or even exclusive for subset A or subset B, which also indicates differences between the polarization process of WT and KO cells.

**Fig. 3:**
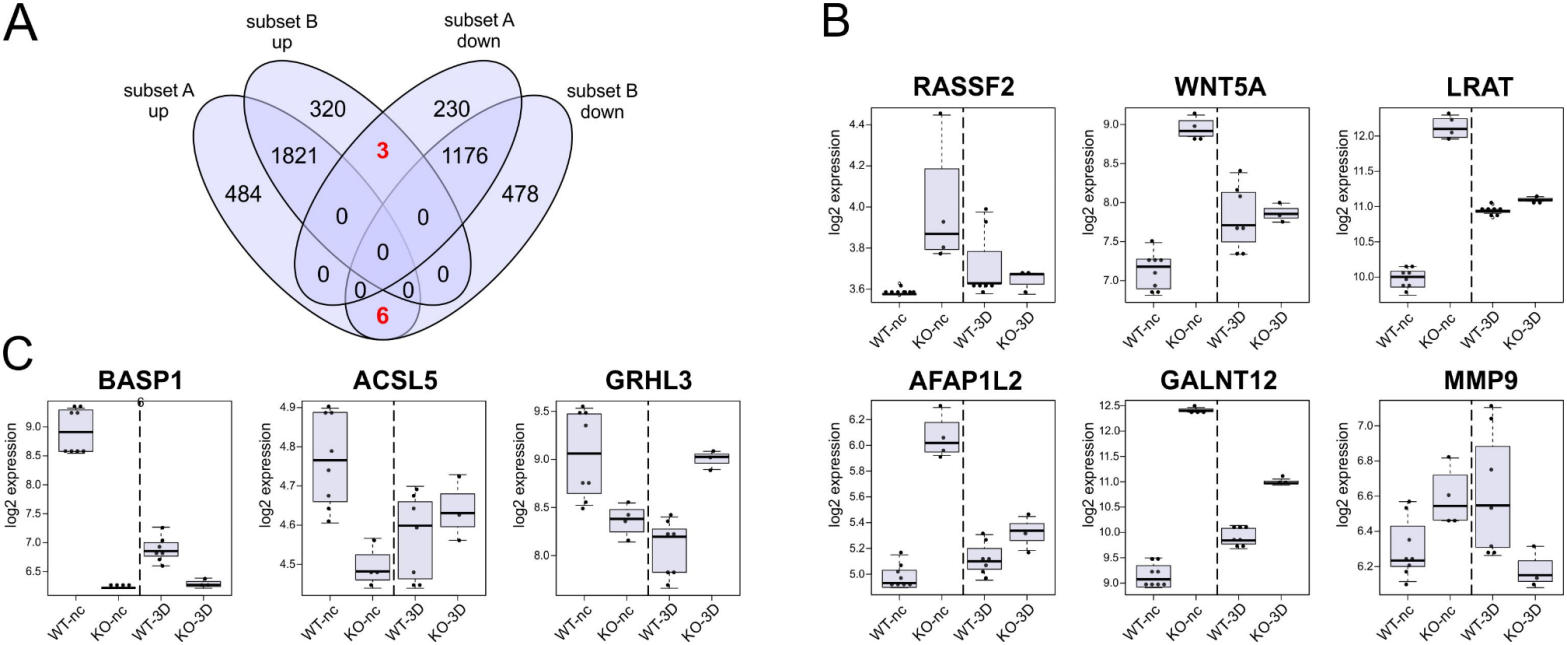
DEGs with an inversed expression profile in WT and *PALS1* KO MDCK cells during cell polarization. **A**: 4-way Venn diagram illustrating overlaps of up- and downregulated DEGs of subsets A and B. The analyses revealed nine DEGs with a “inverse” expression pattern during the transition of nonconfluent (unpolarized) cell to polarized cell, meaning that these genes were upregulated in WT differentiation and downregulated in the KO differentiation process, or vice versa. **B**: Box plot of the six genes that were upregulated WT- and downregulated during differentiation of the KO cells. **C**: Box plot of the three genes that were upregulated in KO- and downregulated during differentiation of the WT cells. WT-nc; KO-nc: expression level of WT or KO cells grown under non-confluent conditions; WT-3D; KO-3D samples: expression levels of WT or KO cells grown as 3D cysts. Each dot represents the expression value of single samples of independent biological replicates

To address this aspect in more detail we also sought for inversely regulated DEGs that were upregulated during the WT differentiation process but downregulated in KO cells and vice versa. For that we applied an intersection analysis using the four groups the up- and downregulated DEG of subset A and subset B (Tab. ST2A, B). The results are summarized in a 4-way Venn diagram (Fig. 3A). Indeed, we identified six DEGs *(RASSF2*, *WNT5A*, *LRAT*, *AFAP1L2*, *GALNT12, MMP9)* that were upregulated in WT differentiation, but downregulated in the analogues process of KO cells (Fig. 3B), whereas, vice versa, the three DEGs *BASP1*, *ACSL5*, and *GRHL3* were downregulated during the WT-nc to WT-3D transition, und upregulated during the differentiation process of cells lacking *PALS1* (Fig. 3C). These inversely regulated DEGs may be indicative of differences between WT and *PALS1*-deficient cells.

### Gene ontology (GO) enrichment studies revealed strong overlaps between the polarization processes of MDCK WT and PALS1-depleted MDCK cells

**Fig. 4:**
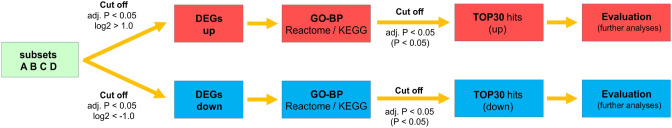
Gene ontology (GO) term enrichment studies and gene over-representation analyses (ORAs) to identify cellular consequences of *PALS1* depletion in MDCK cells. FLOW chart: The individual transcriptomes from non-confluent and 3D cell cultures of WT and *PALS1* KO MDCK cells were first processed and grouped using DEseq2. Subsequently, various comparisons were conducted to obtain contrasts called subset A, B, C, and D, respectively (green box). Only genes that were at least a 2-fold up- or downregulated and adj. *P* < 0.05 (cut-off criterion) were considered. Up- and down-regulated DEGs were then utilized for gene ontology (GO) enrichment studies (category: BP from Biological Process) or gene over-representation analyses (ORAs). Here, predominantly pathways with adjusted p-values *P* < 0.05 were considered (cut-off criterion). The top 30 significant pathways (TOP30) in each case were further evaluated

Differences between WT and *PALS1* KO cells may also be linked to larger groups of moderately regulated genes that are linked to certain biological processes or pathways. To address this issue we took advantage of the *EnrichR* software [35] and analyzed which of the gene ontology (GO) categories *“Biological Processes”* (GO-BP) were associated with the DEGs of subsets A and B. In addition to the GO-BP enrichment studies, we also included over-representation analyses (ORAs) using REACTOME [36], and KEGG [37, 38] pathway databases, to confirm matched biological processes and pathways by alternative databases. For these studies an adj. P below 5% was applied as the major cut-off criterion. In case there were more than 30 matches, the thirty strongest regulated categories or pathways (TOP30), ranked by the lowest adj. P were considered. Finally, these criteria were combined with analyses to identify GO-BP categories or KEGG and REACTOME pathways that were shared or rather exclusive to the DEGs groups of the different subsets (Fig. 4). 

**Fig. 5:**
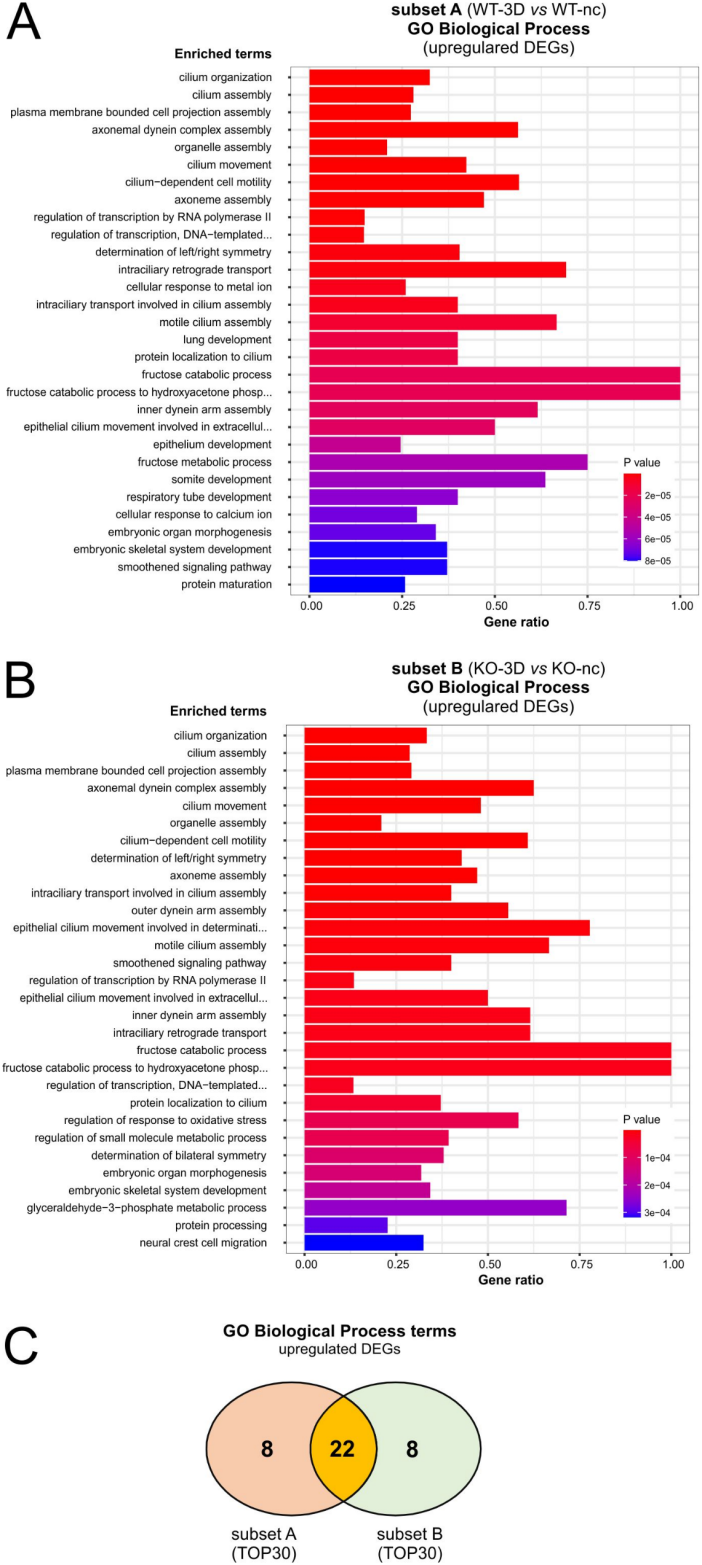
GO-Biological Process term enrichment study of upregulated DEGs associated with subsets A and B. **A** and **B**: EnrichGO analyses: Diagram shows TOP30 hits of a GO-BP enrichment analysis for upregulated DEGs of subset A (A) and subset B (B). **C**: 2-way Venn diagram illustrating shared gene ontology biological process (GO-BP) categories matched by upregulated DEGs of subsets A and B

First, GO-BP enrichment studies linked to the upregulated DEGs of subsets A (Fig. 5A) and subset B (Fig. 5B) were performed. The TOP30 GO-BP categories of both subsets included numerous terms that were linked to the establishment of primary cilia, organ and epithelia development, and some metabolic and transport processes (Fig. 5A, B; Tab. ST4, ST5). Indeed, 22 of the TOP30 GO-BP categories for upregulated DEGs were shared between subsets A and B, and only eight GO-BP were either linked to the WT or the KO-cell associated upregulated DEG-subsets (Fig. 5C). Among the 22 common BO-BP terms were the terms, *cilium organization* (GO:0044782), *cilium assembly* (GO:0060271), *cilium movement* (GO:0003341), or *cilium-dependent cell motility* (GO:0060285). Further shared GO-BP terms were linked to the motility of cilia like the categories *intraciliary retrograde transport* (GO:0035721), *intraciliary transport involved in cilium assembly* (GO:0035735), *motile cilium assembly* (GO:0044458), *protein localization to cilium* (GO:0061512), *inner dynein arm assembly* (GO:0036159), or *axoneme assembly* (GO:0035082), or *axonemal dynein complex assembly* (GO:0070286). In addition, there were also common GO-BP terms that matched biological processes that were associated with *cilia-function*, *like determination of left/right symmetry* (GO:0007368) or *embryonic organ morphogenesis* (GO:0048562). Moreover, some of the GO-BP terms were associated with metabolic pathways including *fructose catabolism* (GO:0006001, GO:0061624) (Fig. 5A, B; Tab. ST4,ST5).

To confirm the GO-BP enrichment studies for upregulated DEGS of subsets A and B we included ORAs using the REACTOME (Tab. ST6,ST7) and KEGG pathway databases (Tab. ST8,ST9). Using adj. P below 5% as threshold led to only four REACTOME matches for the upregulated DEGs of subset A (WT-3D vs. WT-nc) and three for subset B (KO-3D vs. KO-nc). Strikingly, the three REACTOME pathways, *assembly of the primary cilium*, *activation of Smoothened/SMO* and *fructose metabolism* were shared by subsets A and subset B, supporting, first, that most of the matched pathways were shared by WT and KO-cells during polarization, and second, that they were linked to ciliogenesis, epithelia establishment and fructose metabolism. The pathway *intraflagellar transport* of the REACTOME analyses was only significant (adj. *P* < 0.05) for upregulated DEGs of the WT (Tab. ST6,ST7). Using the TOP30 regulated pathways with a *P* < 0.05 instead of an adj. value of *P* < 0.05, confirmed the strong overlap between pathways of WT and KO cells. Using an adj. *P* < 0.05 no KEGG-pathways could be identified (Tab. ST8,ST9).

**Fig. 6:**
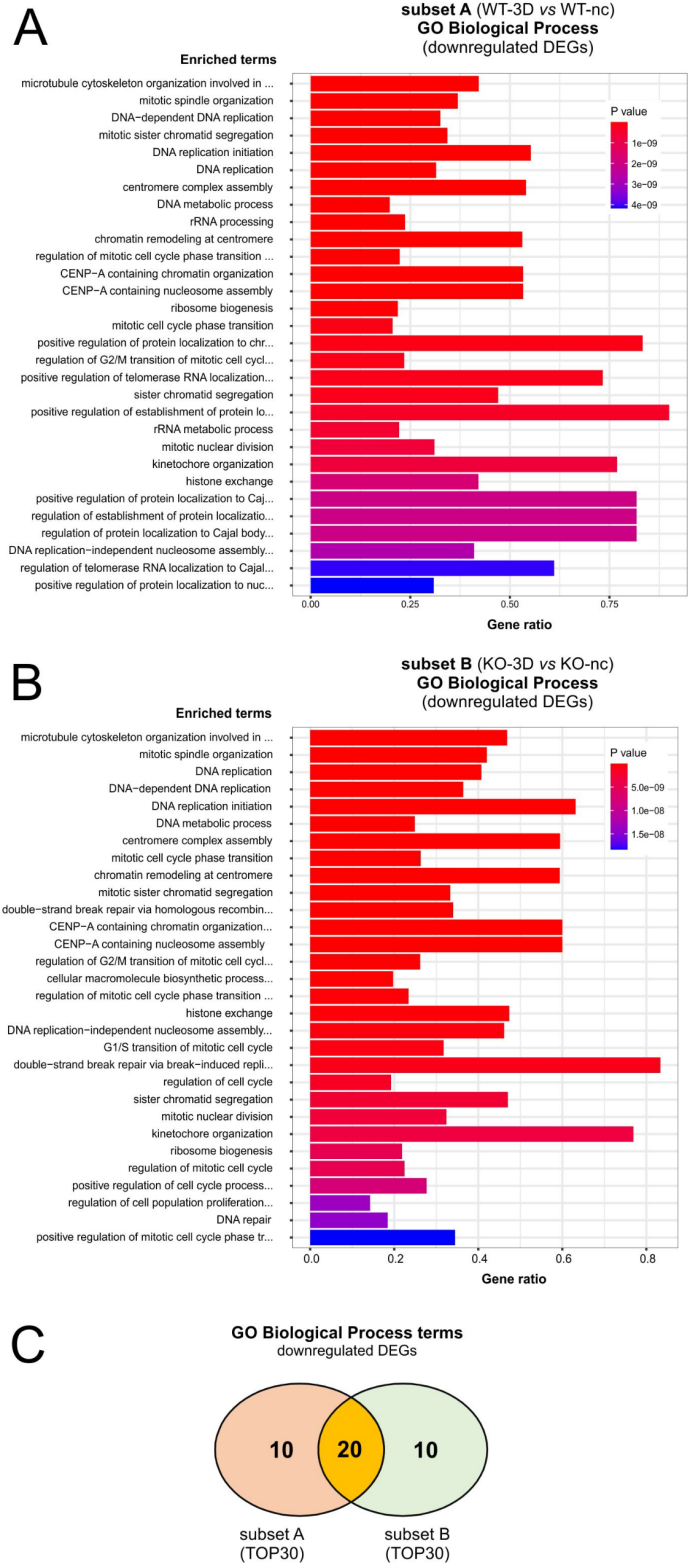
GO-Biological Process term enrichment study of upregulated DEGs associated with subsets A and B. **A** and **B**: EnrichGO analyses: Diagram shows TOP30 hits of a GO-BP enrichment analysis for downregulated DEGs of subset A (A) and subset B (B). **C**: 2-way Venn diagram illustrating shared gene ontology biological process (GO-BP) categories matched by downregulated DEGs of subset A and B

Similar GO-BP term enrichment studies and ORAs were performed for downregulated DEGs of subset A (Fig. 6A; Tab. ST10) and subset B (Fig. 6B; Tab. ST11). Strikingly, here we observed an overlap of 20 of the TOP30 GO-BP terms (Fig. 6C, Tab. ST10,ST11). The common GO-BP predominantly matched categories that have been connected to mitosis and cell cycle-progression for example *microtubule cytoskeleton organization involved in mitosis* (GO:1902850), *mitotic spindle organization* (GO:0007052), *regulation of mitotic cell cycle phase transition* (GO:1901990), *mitotic cell cycle phase transition* (GO:0044772), *regulation of G2/M transition of mitotic cell cycle* (GO:0010389), or ones that were connected to centromere assembly and chromosome segregation processes, like *centromere complex assembly* (GO:0034508), *chromatin remodeling at centromere* (GO:0031055), *CENP-A containing chromatin organization* (GO:0061641), *CENP-A containing nucleosome assembly* (GO:0034080), *kinetochore organization* (GO:0051383), or *sister chromatid segregation* (GO:0000819). The GO-BP terms also included categories linked to DNA replication and DNA metabolism, like *DNA-dependent DNA replication* (GO:0006261), *mitotic sister chromatid segregation* (GO:0000070), *DNA replication initiation* (GO:0006270), *DNA replication* (GO:0006260), *DNA replication-independent nucleosome assembly* (GO:0006336), or *DNA metabolic process* (GO:0006259) (Fig. 6; Tab. ST10,ST11).

As for the upregulated DEGs, we included also ORAs with the REACTOME and KEGG pathways. The studies with REACTOME database showed a high degree of common pathways. Most of them were linked to different phases of the cell cycle, the separation of sister chromatids, or to DNA replication. These hits confirmed the results found by the GO-BP enrichment studies. In addition, we found pathways that were connected to Rho-GTPase-associated signaling (R-HSA-195258, R-HSA-194315) (Tab. ST12,ST13). GTPases of the Rho family and their regulators play a key role in the control of actin dynamics and also during the polarization process of MDCK cells [39, 40]. ORAs using KEGG pathways (Tab. ST14,ST15) revealed several pathways that - in contrast to the upregulated DEGs - matched adj. *P* < 0.05. Among the 23 common KEGG pathways were numerous pathways linked to the cell cycle and DNA-replication that confirmed the findings using GO-BP terms and the REACTOME database but also matches linked to *Hippo*- and *TGF-beta* signaling.

Together, the GO-BP enrichment studies and ORAs with the REACTOME and KEGG databases (Figs. 5 and 6; Tab. ST2-15) confirmed the observations performed with a focus on the DEGs alone (Figs. 2 and 3) and demonstrated a striking similarity between the differentiation processes of WT and *PALS1* KO MDCK cells. However, the studies also provide evidence for small, but significant differences particularly in quantitative aspects (due to different ranking and adj. P of identical matched terms) and qualitative parameters (evident by the presence or absence of some terms or pathways linked only to the WT or KO DEGs analyses).

### Comparison of KO versus WT DEGs of cells grown in 3D cysts or under non-confluent conditions

**Fig. 7:**
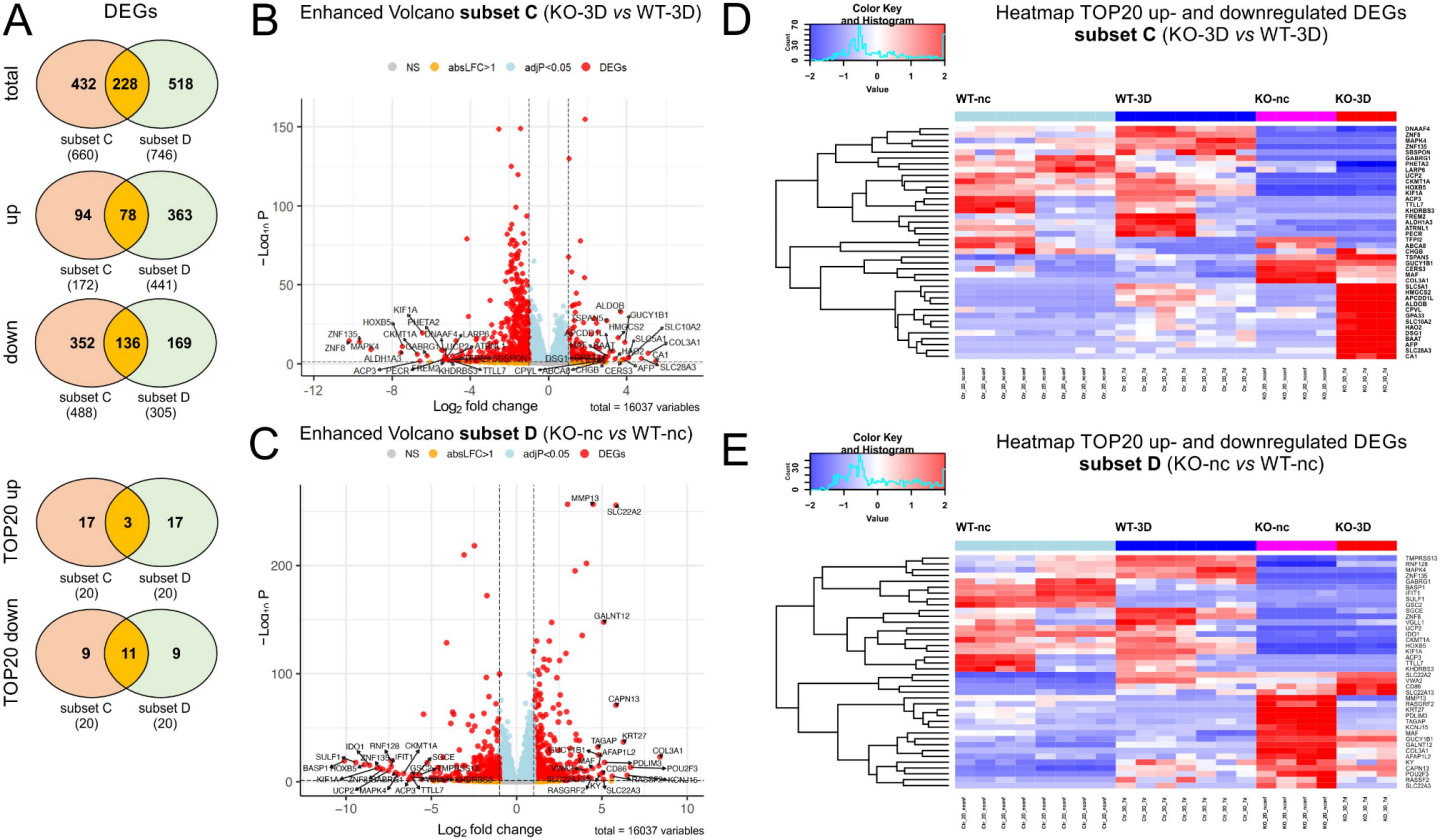
Summary of DEGs of WT and *PALS1* KO MDCK cell grown in 3D cyst cultures. **A**: Venn diagrams illustrating overlaps of total (upper diagram), upregulated (middle diagram) and downregulated DEGs of subsets C and D. The Venn diagrams of TOP20 up- and downregulated genes, overlapping in both subsets C and D. **B** and **C**: Volcano plots of DEGs of *subsets C* (B) and *subset D* (C), with annotated TOP20 up- and downregulated genes. y-axis: -log_10_, adj. P, x-axis: log_2_ fold change. DEGs are colored red and the TOP20 up- and down-regulated (by log-fold change) DEGs are labeled. Blue: genes with adj. *P* < 0.05. Yellow: genes with absolute log_2_-fold change > 1. **D** and **E**: Heatmap of TOP20 upregulated (D) and downregulated DEGs (E) showing the difference of relative gene expression levels in WT and KO cell grown 3D cyst (subset C) or in non-confluent monolayers (subset D)

To identify whether *PALS1*-KO-induced cell polarization defects and malformed cell-cell junctions were associated with an altered gene expression profiles we also included a *direct* comparison of the WT and KO 3D cell grown in 3D cultures or in non-confluent monolayers. The comparison of the cyst transcriptomes led to 660 DEGs with 172 being up- and 488 down regulated, when normalized to the expression level of the KO cells (Fig. 1; subset C, Tab. ST2C). The gene expression profiles of non-confluent WT and *PALS1* KO cells resulted in 746 total DEGs of which 441 were up- and 305 down-regulated. The direct comparison of mRNA profiles derived from 3D cyst of WT and *PALS1* KO cells resulted in 660 DEGs of which 172 were up- and 342 down-regulated (Fig. 1; subset D, Tab. ST2D). Most of the DEGs of subsets C and D were either exclusively for 3D cysts (44% equivalent to 432 DEGs) or non-confluent cells in (36.7% equivalent to 518 DEGs), only 228 DEGs (19.4%) were shared by non-confluent and 3D cells (Fig. 7A; Tab. ST16). When the up- and downregulated DEGs were considered separately, the common DEGs accounted for 14.6% for the upregulated and 20.7% for the downregulated DEGs. Thus, most DEGs were specific to either non-confluent or 3D stages, with only a minor fraction (about 20%) regulated independently of their growth conditions (Fig. 7A). The Volcano plots summarize the DEG analyses for subsets C and D (Fig. 7B, C), respectively. The strongest TOP20 up- and down regulated genes that were also listed in heatmaps (Fig. 7D, E).

Next, we analyzed which of the most up- and downregulated DEGs (TOP20 genes) were exclusive or shared by 3D cysts and non-confluent cells (Tab. ST16). Only three DEGs *(MAF*, *GUCY1B1*, *COL3A117)* of the most strongly up-regulated (TOP20 up) DEGs were shared between subsets C and D. The majority, 17 DEGs, were exclusively for the 3D stages *(ABCA8*, *CPVL*, *GPA33*, *DSG1*, *CHGB*, *TSPAN5*, *APCDD1L*, * BAAT*, *HAO2*, *HMGCS2*, *ALDOB*, *CERS3*, *AFP*, *SLC5A1*, *SLC10A2*, *SLC28A3*, *CA1)*, or for the non-confluent cells *(VWA2*, *SLC22A13*, *RASGRF2*, *MMP13*, *TAGAP*, *AFAP1L2*, *KY*, * CD86*, *SLC22A3*, *GALNT12*, *PDLIM3*, *RASSF2*, *CAPN13*, *SLC22A2*, *KRT27*, *KCNJ15*, *POU2F3)*. From the TOP20 down-regulated DEGs eleven were shared by subset C and D *(ZNF8*, *ZNF135*, *MAPK4*, *HOXB5*, *CKMT1A*, *ACP3*, *KIF1A*, *GABRG1*, *UCP2*, *KHDRBS3*, *TTLL7*). Nine were either exclusive for the 3D conditions *(ALDH1A3*, *PHETA2*, * FREM2*, *DNAAF4*, *PECR*, *LARP6*, *TFPI2*, *ATRNL1*, *SBSPON)*, or the non-confluent cells *(BASP1*, *SULF1*, *IDO1*, *IFIT1*, *RNF128*, *MAPK4*, *GSC2*, *SGCE*, *TMPRSS13*, *VGLL1)*.

**Fig. 8:**
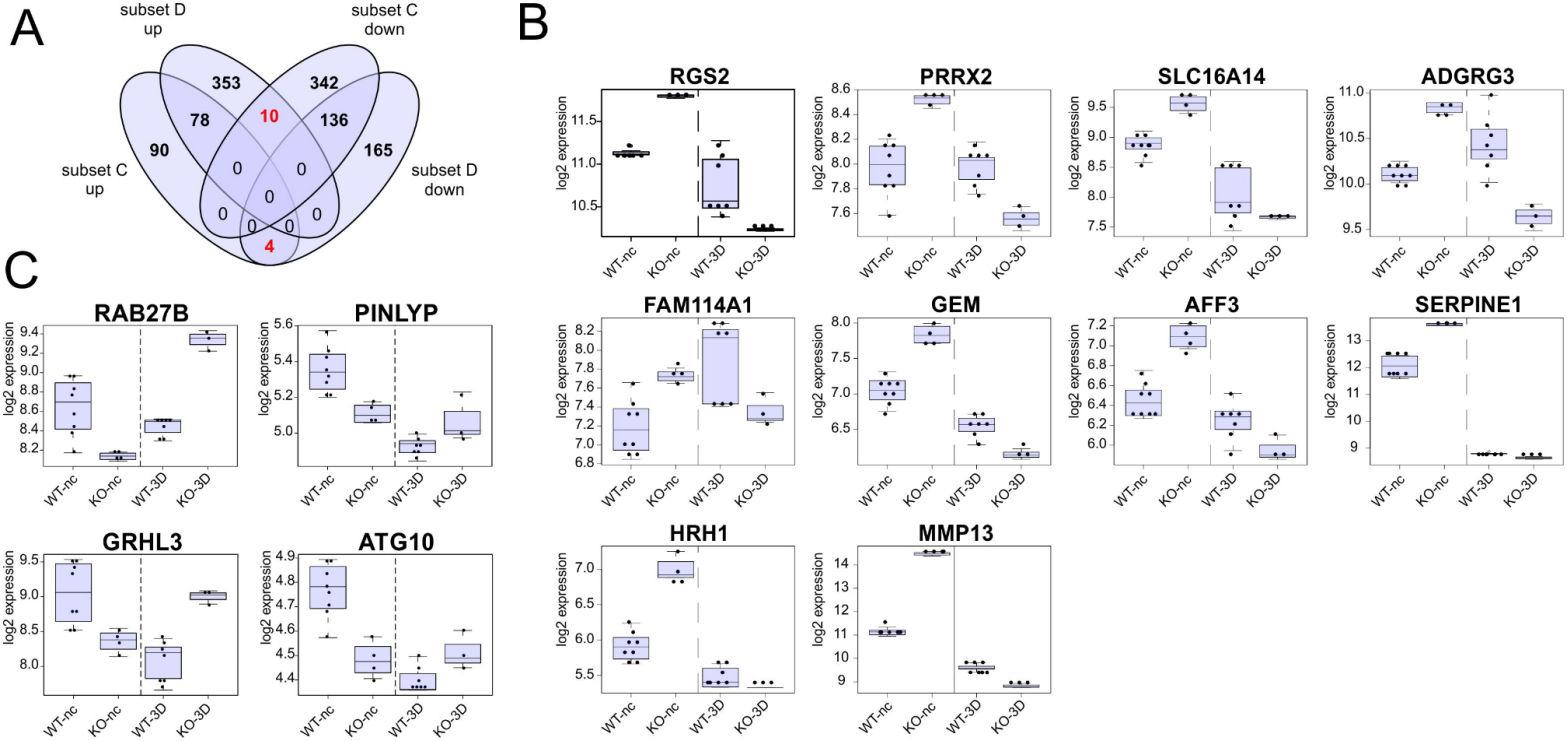
DEGs with an inversed expression profile in non-confluent monolayers and 3D cysts. **A**: 4-way Venn diagram illustrating overlaps of up- and downregulated DEGs of subsets C and D. The analyses revealed 14 DEGs with a “reciprocal” expression pattern meaning that they were among the upregulated DEGs of subset C (WT vs. KO at 3D stage) and downregulated in subset D (WT vs. KO at non-confluent stage), or vice versa. **B**: Box plot of the ten DEGs that were expressed higher in KO than in WT cells during non-confluent conditions but less expressed in the KO than in WT cells at the 3D stage. **C**: Box plot of the four DEGs that were expressed higher in WT than in KO cells during non-confluent conditions but less expressed in the WT than in KO cells at the 3D stage. WT-nc; KO-nc: expression level of WT or KO cells grown under non-confluent conditions; WT-3D; KO-3D samples: expression levels of WT or KO cells grown as 3D cysts. Each dot represents the expression value of a single sample of independent biological replicates

Similar, as done for subsets A and B, we used the four groups of up-and downregulated DEGs of cells grown in 3D cysts (subset C) and non-confluent cells (subset D) to seek for inversely regulated DEGs (Fig. 8). This resulted in 14 DEGs of which ten *(RGS2*, *PRRX2*, *SLC16A14*, *ADGRG3*, *FAM114A1*, *GEM*, *AFF3*, *SERPINE1*, *HRH1*, *MMP13)* were upregulated in non-confluent KO cells but down regulated at the 3D stage, meaning that these genes are expressed higher in the WT than in KO cysts. Vice versa, four DEGs *(RAB27B*, *PINLYP*, *GRHL3*, *ATG10)* were found to be downregulated at non-confluent KO cells but upregulated in KO cysts cells (Fig. 8).

### Gene ontology (GO) enrichment studies of MDCK WT and PALS1-depleted MDCK cells grown in nonconfluent monolayers or 3D cyst cultures

**Fig. 9:**
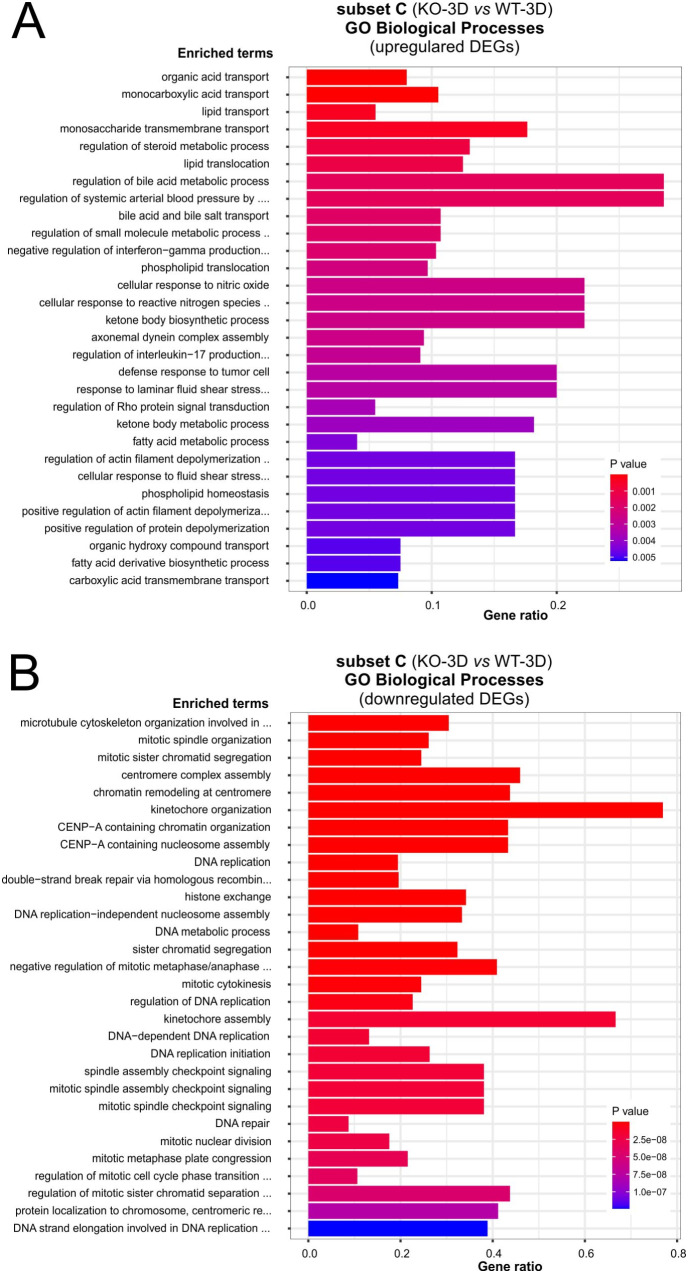
Summary of matched GO Biological Process categories of subset C (KO-3D vs. WT-3D). **A** and **B**: EnrichGO analyses of subset C: Diagram shows TOP30 hits of a GO-BP enrichment analysis for up- (A) and down regulated DEGs (B) of subset C (KO-3D vs. WT-3D), respectively. (For upregulated DEGs, only the terms *organic acid transport* and *monocarboxylic acid transport* showed an adj. *P* < 0.05)

We first focused on GO-BP term enrichment studies for up- and downregulated DEGs (adj. *P* < 0.05) of subset C. GO-BP enrichment studies for the up-regulated DEGs resulted in only two matches: *organic acid transport* (GO:0015849) and *monocarboxylic acid transport* (GO:0015718), most probably due to a rather small group of 172 DEGs (Fig. 9A; Tab. ST17). For the downregulated DEGs GO-BP enrichment studies we identified more than 150 hits (Fig. 9B; Tab. ST18). The matches of the TOP30 list resembled the GO-BP hits found for subsets A and B (Tab. ST3, ST4 and ST10, ST11) and contained numerous hits, again predominantly linked to mitotic processes such as microtubule-associated processes during mitosis including spindle formation, centromere/kinetochore assembly, the separation of sister chromatids, or DNA replication and repair (Fig. 9B; Tab. ST18). For the upregulated DEGs (441) of subset D we identified 16 GO-terms (Tab. ST19). These pathways include some GO terms linked to organization of the extracellular matrix, and integrin-mediated signaling pathways (e.g. GO:0043062, GO:0030198, or GO:0007229). Downregulated DEGs (305) of subset D matched seven GO-terms (Tab. ST20) and included pathways linked to cytokines (e.g. GO:0019221, GO:0071357, GO:0060337). An intersection study with GO-BP terms linked to up- and downregulated DEGs showed no overlaps between subsets C and D. Similar studies using REACTOME (Tab. ST21-ST24) pathways strongly confirmed these results of the GO-BP enrichment studies, there were no overlaps between pathways of subsets C and D. For subset C upregulated DEGs matched 10 KEGG pathways, including *“cell adhesion molecules”* (Tab. ST25) Downregulated DEGs matched 18 pathways and include cell cycle and TNF signaling pathway (Tab. ST26). The ORAs studies with DEGs of Subset D matched six KEGG pathways for up – and nine for downregulate DEGs (Tab. ST27, ST28). Of note, upregulated DEGs of subset D matched *Hippo signaling* that has linked to PALS1 and the Crumbs complex before [13, 41]. In contrast to the GO-BP term and REACTOME studies KEGG studies identified two over lapping pathways, one for up (*Mucin type O-glycan analysis*) and one for downregulated DEGs (*TNF signaling pathway*).

### A minor fraction of DEGs linked the 3D cyst stage overlap with genes regulating cell-cell contact formation, cell polarization or the interactions with the extracellular matrix

As mentioned, deletion of *PALS1* (or *PATJ*) is associated with a reduced distribution of TJ proteins along the bi-cellular cell contacts [12, 23]. This phenotype is only observed in apicobasally polarized but not in non-confluent, unpolarized cells. Polarization in turn also depends on cell interactions with the extracellular matrix (ECM). To include these aspects in our analysis, we therefore investigated how the expression levels of genes that *(i)* encode for junctional proteins, *(ii)* control the establishment or maintenance of cell polarity (consisting of), and *(iii)* mediate cell-substrate interactions matched with DEGs identified for the 3D stage (3D-KO versus 3D-WT).

For the gene list “cell junctions”, we combined genes listed in the GO terms *apical junction complex* (GO:0043296), *tight junction* (GO:0070160) and *adherens junction* (GO:0005912), resulting in 296 genes (Tab. ST29). The gene list of “cell polarity” based on the GO-term *establishment or maintenance of cell polarity* (GO:0007163) and consists of 230 genes; (Tab. ST29). For the gene list substrate interactions, called “cell-ECM” we used GO terms *cell-substrate adhesion* (GO:0031589), *cell-matrix adhesion* (GO:0007160) and *cell adhesion mediated by integrin* (GO:0033627). This gene list consists of 386 genes.

**Fig. 10:**
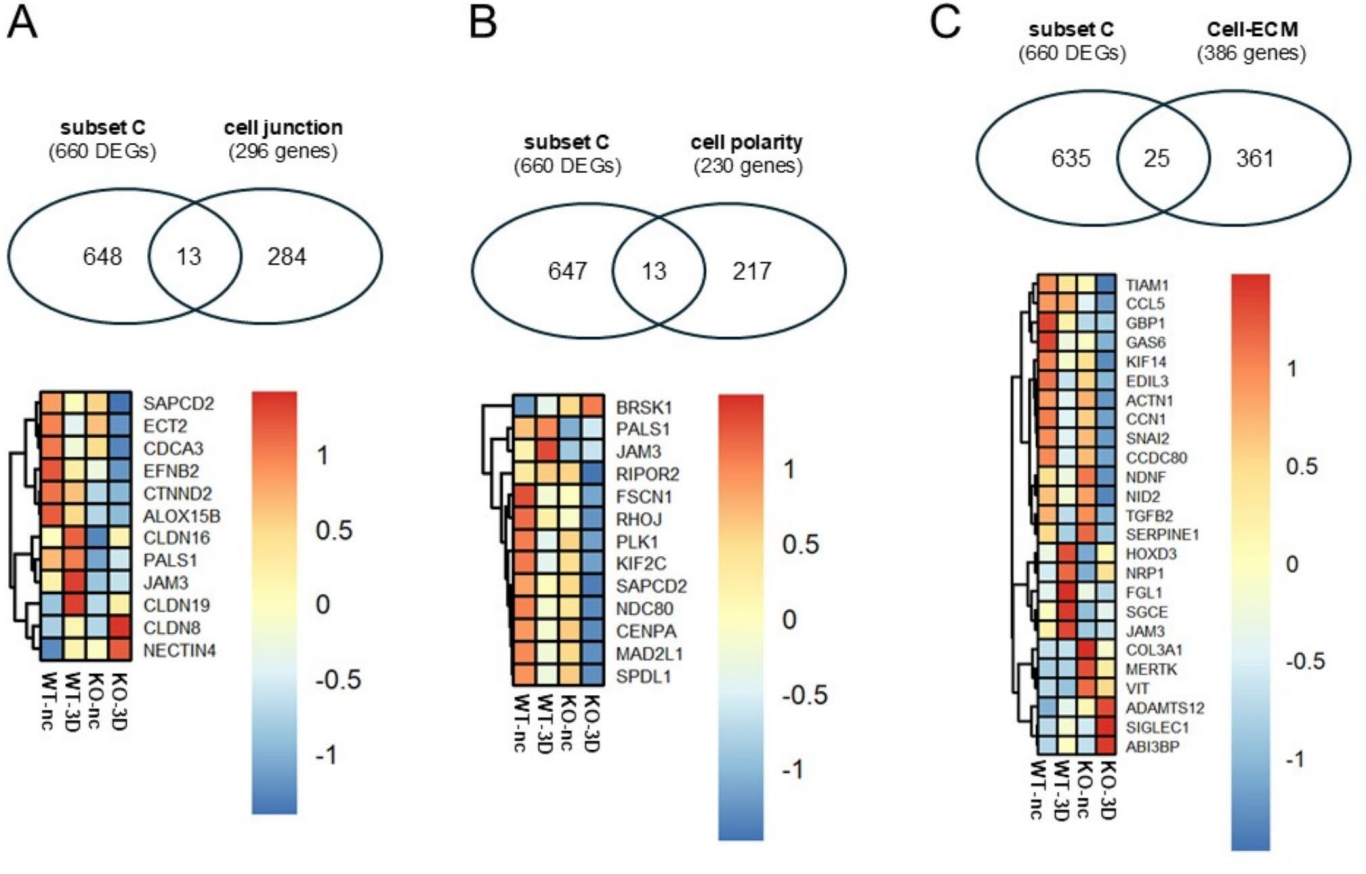
DEGs linked with 3D cyst stage to genes associated with cell-cell contact, cell polarization, and cell-substrate interactions. **A**: Upper 2-Venn diagram: Overlap of DEGs of subset C *(KO-3D versus WT-3D)* with genes encoding junctional proteins (296 genes; Tab. ST29). *Lower Heatmap*: common genes of both groups. **B**: Upper 2-Venn diagram: Overlap of DEGs of subset C *(KO-3D versus WT-3D)* with genes encoding for polarity proteins (230 genes; Tab. ST29). *Lower Heatmap*: common genes of both groups. **C**: Upper 2-Venn diagram: Overlap of DEGs of subset C with genes encoding for proteins involved in cell-substrate interactions (386 genes; Tab. ST29). *Lower Heatmap*: common genes of both groups

The intersection of the subset C (660 DEGs) with genes encoding cell junction proteins comprises twelve genes (Fig. 10A). Six of these genes were already DEGs in non-confluent cells, (*CTNND2, PALS1, CLDN16, JAM3, ALOX15B, NECTIN4*), including as expected *PALS1*. Six genes were exclusive for the 3D stage, with five being down (*CLDN19*, *SAPCD2*, *ECT2*, *CDCA3*, *EFNB2*) and one *(CLDN8)* being upregulated in KO 3D cysts cells (Fig. 10A).

The intersection of subset C with genes involved in cell polarity includes 13 genes (Fig. 10B). Here five genes, including PALS1, were also found to DEGs in non-confluent cells *(PALS1*,*JAM3*, *FSCN1*, *RHOJ*, *BRSK1)*. Seven genes *(PLK1*, *NDC80*, *SPDL1*, *MAD2L1*, *RIPOR2*, *SAPCD2*, *KIF2C*, *CENPA)* were specific for 3D cysts cells and showed a reduced expression (Fig. 10B).

Moreover, we identified 25 DEGs from subset C that were also associated with cell-extracellular matrix (ECM) interactions (Fig. 10C). From these genes, 12 genes *(SGCE*, *GBP1*, *JAM3*, *NRP1*, *HOXD3*, *EDIL3*, *GAS6*, *SERPINE1*, *VIT*, *ADAMTS12*, *MERTK*, *COL3A1)* were also DEGs of non-confluent cells. 13 DEGs were only differentially expressed at the 3D cyst in which the basal membrane mediates interactions with the ECM (Matrigel). Eleven of these 13 genes *(NID2*, *ACTN1*, *CCL5*, *FGL1*, *KIF14*, *TIAM1*, *NDNF*, *CCN1*, *TGFB2*, *SNAI2*, *CCDC80)* were down- and two *(SIGLEC1, ABI3BP)* upregulated at the cyst stage (Fig. 10C). Together these data demonstrate that only small fractions of subset C overlap with genes involved in regulating cell-cell contact formation, cell polarization, or interactions with the extracellular matrix.

## Discussion

The loss of PALS1 caused an increased formation of cysts with no, or multiple lumens [12], similar to what has been previously shown by Margolis and colleagues [10, 11]. In addition, apicobasally polarized *PALS1* KO cells showed reduced presence of TJ components (for example ZO-1) and the PALS1 binding partner PATJ along bi-cellular junctions. In 3D cysts, the altered TJ protein distribution was also accompanied by strong permeability defects, indicating that PALS1 serves as a regulator of the lateral distribution of TJ proteins [12]. So far this phenotype of *PALS1* depleted cells is primarily linked to the protein and cellular biological levels. To determine if and how *PALS1* depletion might be associated with transcriptional changes (mRNA level), we compared transcriptomes of wildtype MDCK cells with *PALS1* KO cells in early immature stage (non-confluent) and mature differentiated ones (3D cysts).

The direct comparison of the transcriptomes of non-polarized and polarized cells revealed the differential expression of more than 3,700 genes, both in WT and KO cells. The high number of DEGs demonstrates that the transition from non-confluent cells to polarized epithelia was associated with significant changes in mRNA expression profiles. However, most of these DEGs (> 3,000 DEGs) were shared between WT and KO cells. Even for the most up- or downregulated genes, there were substantial overlaps between WT and KO cells. This suggests that the epithelial polarity program process, which governs the transition from non-polarized mitotically active cells to polarized epithelial cells mainly followed similar routes in WT and KO cells [42, 43].

The development of cellular phenotypes does not necessarily have to be associated with the most strongly regulated genes. Instead, it is possible that moderate regulation of larger gene groups that work together could initiate cellular phenotypes or have a significant impact on shaping them. Indeed, for upregulated DEGs, the GO-term enrichment studies and ORAs using REACTOME and KEGG pathways databases elucidated ciliogenesis as one key event for the differentiation of non-confluent cells into highly polarized epithelia. For downregulated DEGs, we predominantly identified numerous GO-terms and pathways linked to the regulation of mitosis, in particular processes controlling spindle formation or the assembly of the centromere/kinetochore. Furthermore, the ORAs also matched several signaling pathways, including WNT-, Hippo-, Hedgehog, and mTOR signaling which have not only been linked to ciliogenesis and/or ciliopathies before [44–47], but also directly to PALS1 [13, 41, 48–50]. The large number of overlapping DEGs as well as the results from GO-BP enrichment studies and ORAs using subsets A and B confirm the striking similarities between WT and KO cells and suggest that the majority of differentially regulated genes and their associated pathways are linked to the polarization process of MDCK cells and not to the loss of PALS1.

A small group of genes that were not among the TOP regulated DEGs were inversely regulated. The DEGs were upregulated during the WT and downregulated during the KO differentiation process. They included *RASSF2*, *WNT5A*, *LRAT*, *AFAP1L2*, *GALNT12*, *and MMP9*. Vice versa, *BASP1*, *ACSL5*, *and GRHL3* were highly expressed during the differentiation of PALS1 depleted cells but decreased during the differentiation of WT cells. Remarkably, some of these genes provide links to Wingless (WNT)-associated signaling pathways. *WNT5A* (Wnt family member 5 A) for example plays a role in the Wingless/planar polarity (WNT/PCP) signaling pathway, which is crucial for organ and epithelial development [51, 52]. Disturbances in this pathway have been linked to various genetic disorders, including several renal ciliopathies (Benzing et al., 2007; Santoni et al., 2020, and references therein). In contrast to the WT maturation process, expression levels the transcription *GRHL3 (grainyhead like transcription factor 3)* increase during KO differentiation *GRHL3* is a member of the highly conserved *grainy head* family of TFs that play a significant role development and remodeling of epithelia [53]. Interestingly, *GRHL3* was also identified as a crucial downstream effector of the WNT/β-catenin signaling pathway, which controls epithelial differentiation [54]. Thus, the differential expression of these genes might be a hint that PALS1 depletion is somehow linked to an imbalanced or altered WNT-signaling. Together, our studies, focusing on the comparison between non-confluent and cells grown in cysts point in the direction that PALS1 serves not as an essential element of polarization and differentiation but rather as a modulator of these processes.

The second focus of our work was the direct comparison of the transcriptional profiles of WT and KO cells to clarify which DEGs were differentially regulated per se and thus independently of the maturation status, and which DEGs were only present in the polarized stages where the phenotype was found. This direct comparison between WT and KO revealed two interesting aspects: first, in both *WT-versus-KO* comparisons, there were significantly fewer DEGs than in the comparison of differentiation processes. Second, the overlap between these two subsets was only about 15–20% (228 DEGs), meaning that the majority of DEGs were either specific to non-confluent cells or cells grown at 3D stages. Due to the lower number of DEGs, fewer pathways and biological processes were found in the GO-term enrichment and ORA studies. However, overall, these GO-term studies and ORAs confirm that there were more differences than similarities in the direct comparison between WT and KO cells. DEGs that were differentially regulated per se and independently of maturation status may be involved in regulating cell proliferation in immature cells and function establishment and maintenance in differentiated cells. This means that although phenotypes appear at the 3D stage, the origin may be at early-stage differences, for example by genes that drive the maturation process. Another possibility is that some of these common DEGs have different functional roles in the two stages. The extent to which this applies to the identified DEGs need further in-depth investigation, however the identified transcription factors *(ZNF8*, *ZNF135*, *HOXB5*, or *MAF)* may be particularly interesting in this context.

The GO-BP term enrichment studies and ORAs identified only two pathways for the upregulated DEGs of KO cells from the 3D cysts, but numerous GO-BP terms for the downregulated DEGs. The majority of these terms were associated with mitosis, the transition between different cell cycle checkpoints, chromosome distribution, the assembly of centrosomes/kinetochores, or DNA replication and repair. Overall, this suggests that while general cell polarization was not completely disrupted, PALS1-depletion resulted in modifications of important associated signaling pathways.

The loss of PALS1 and PATJ in polarized MDCK cells is associated with reduced levels of TJ proteins (e.g. ZO-1, ZO-2, Occludin) in the bi-cellular junctions. The distribution along the bi-cellular junction in MDCK cells is controlled by a recently identified wetting process that ensures the even distribution of TJ proteins. This process depends on PALS1 and PATJ and most likely by further indirect and direct binding partners. An interesting question, therefore, is whether PALS1 depletion affects the expression levels of genes coding for these and other cell junction proteins. In the intersection analyses, we only identified a few genes. However, it remains unclear whether these changes are also present at the protein level and, if so, what role they play for TJs in MDCK 3D cysts.

Interestingly, our studies and others have shown that the loss of PALS1 is associated with a loss of PATJ at the protein level [10, 12, 43]. Notably, here we show, that this correlation does not exist at the transcriptional level, suggesting that this PALS1-PATJ correlation is only present at the protein level. Furthermore, among 230 genes associated with the establishment and maintenance of apicobasal cell polarity, very few were found to be DEGs in the comparisons between WT and KO. This indicates that PALS1 does not directly influence the transcription of these genes. A similar small overlap was observed for key genes involved in cell-substrate/ECM interactions. Together this confirmed that PALS1 acts as modulator of these processes.

However, our study has some limitations. First, the analyses assume that the gene repertoire and their associated gene functions are evolutionarily conserved in mammals, particularly in humans and dogs, and therefore largely overlap. Second, the mRNA profiles in MDCK cells may not be representative for all cell types of renal epithelia. And third, it is not known whether the identified changes in transcriptional profiles are directly induced by *PALS1* knockout or if they are secondary effects due to affected cell polarization of *PALS1*-KO cells.

## Conclusion

In this study, we compared the transcriptomes of non-polarized and polarized cells of WT and KO cells in these stages. Our results demonstrate for the first time that the disruption of cell polarity and the lateral misdistribution of tight junction proteins caused by *PALS1* depletion is associated with changes in gene expression patterns and corresponding biological functions and pathways. Whether these changes are caused by alterations in early stages or rather by adaptations, as secondary consequences of PALS1 loss in 3D stages, remains to be analyzed in further cell biology studies.

## Electronic supplementary material

Below is the link to the electronic supplementary material.

Supplementary Material 1

Supplementary Material 2

Supplementary Material 3

Supplementary Material 4

Supplementary Material 5

Supplementary Material 6

Supplementary Material 7

Supplementary Material 8

Supplementary Material 9

Supplementary Material 10

Supplementary Material 11

Supplementary Material 12

Supplementary Material 13

Supplementary Material 14

Supplementary Material 15

Supplementary Material 16

Supplementary Material 17

Supplementary Material 18

Supplementary Material 19

Supplementary Material 20

Supplementary Material 21

Supplementary Material 22

Supplementary Material 23

Supplementary Material 24

Supplementary Material 25

Supplementary Material 26

Supplementary Material 27

Supplementary Material 28

Supplementary Material 29

Supplementary Material 30

Supplementary Material 31

Supplementary Material 32

Supplementary Material 33

## Data Availability

The materials used during the current study are available from the corresponding author on reasonable request. The RNASeq data are deposited in the GEO public database: https://www.ncbi.nlm.nih.gov/geo ID: GSE264311.
