# Supplementary material for: PALS1-dependent modulations of mRNA profiles in MDCK II cells grown in non-confluent monolayers and three-dimensional cysts
Source: BMC Genom Data. 2024 Nov 29;25:102. doi: 10.1186/s12863-024-01284-0 (PMC11607895; doi:10.1186/s12863-024-01284-0)
Supplement: Supplementary file 33 — Supplementary Material 33 [file 12863_2024_1284_MOESM33_ESM.pdf]

Supplemental Data

PALS1-dependent modulations of mRNA profiles in MDCK II cells grown in non-confluent monolayers and 3-dimensional cysts.

by

Klaus Schughart[1, 2*], Annika Möller-Kerutt[3], Verena Höffken[3], Pavel Nedvetsky[4], Ann-Christin Groh[3], Daniela Anne Braun[3], Hermann Pavenstädt[3], and Thomas Weide[3]

[1)] Institute of Virology Münster, University of Münster, 48149 Münster, Germany.

[2)] Department of Microbiology, Immunology and Biochemistry, University of Tennessee Health Science Center, Memphis, TN 38103, U.S.A.

[3)] University Hospital of Münster (UKM), Internal Medicine D (MedD), Dept. Molecular Nephrology, Münster, 48149, Germany

[4)] University Hospital of Münster (UKM), Internal Medicine D (MedD), Dept. Medical Cell Biology, Münster, 48149, Germany

Corresponding author:

Thomas Weide

University Hospital of Münster (UKM), Internal Medicine D (MedD), Dept. Molecular Nephrology, Münster, 48149, Germany

Email: weidet@uni-muenster.de

Table Of Content Supplemental Data

Supplemental Figures

Fig. SF1:	Preparation of mRNA samples for transcriptome analyses.

Supplemental Tables

Table ST1	MDCK transcriptomes of wildtype, PALS1 knockout and the starting MDCK cell pool.
Table ST2A	Differential expressed genes (DEGs) of subset A (WT-3D vs WT-nc)
Table ST2B	DEGs of subset B (KO-3D vs KO-nc)
Table ST2C	DEGs of subset C (KO-3D vs WT-3D)
Table ST2D	DEGs of subset D (KO-nc vs WT-nc)
Table ST03	Shared DEGs of subset A and subset B
Table ST04	Gene ontology biological processes (GO-BP) enrichment analysis for upregulated DEGs of subset A
Table ST05	GO-BP enrichment analysis for upregulated DEGs of subset B
Table ST06	REACTOME analysis for upregulated DEGs of subset A
Table ST07	REACTOME analysis for upregulated DEGs of subset B
Table ST08	KEGG analysis for upregulated DEGs of subset A
Table ST09	KEGG analysis for upregulated DEGs of subset B
Table ST10	GO-BP enrichment analysis for downregulated DEGS of subset A
Table ST11	GO-BP enrichment analysis for downregulated DEGs of subset B
Table ST12	REACTOME analysis for downregulated DEGs of subset A
Table ST13	REACTOME analysis for downregulated DEGs of subset B
Table ST14	KEGG analysis for downregulated DEGs of subset A
Table ST15	KEGG analysis for upregulated DEGs of subset B
Table ST16	Shared DEGs of subset C and subset D
Table ST17	GO-BP enrichment analysis for upregulated DEGs of subset C
Table ST18	GO-BP enrichment analysis for downregulated DEGs of subset C.
Table ST19	GO-BP enrichment analysis for upregulated DEGs of subset D
Table ST20	GO-BP enrichment analysis for downregulated DEGs of subset D.
Table ST21	REACTOME analysis for upregulated DEGs of subset C
Table ST22	REACTOME analysis for downregulated DEGs of subset C
Table ST23	REACTOME analysis for upregulated DEGs of subset D
Table ST24	REACTOME analysis for downregulated DEGs of subset D
Table ST25	KEGG analysis for upregulated DEGs of subset C
Table ST26	KEGG analysis for upregulated DEGs of subset C
Table ST27	KEGG analysis for upregulated DEGs of subset D
Table ST28	KEGG analysis for upregulated DEGs of subset D
Table ST29	Gene lists: cell junction, cell polarity, and cell-ECM interaction

Supplemental Figures

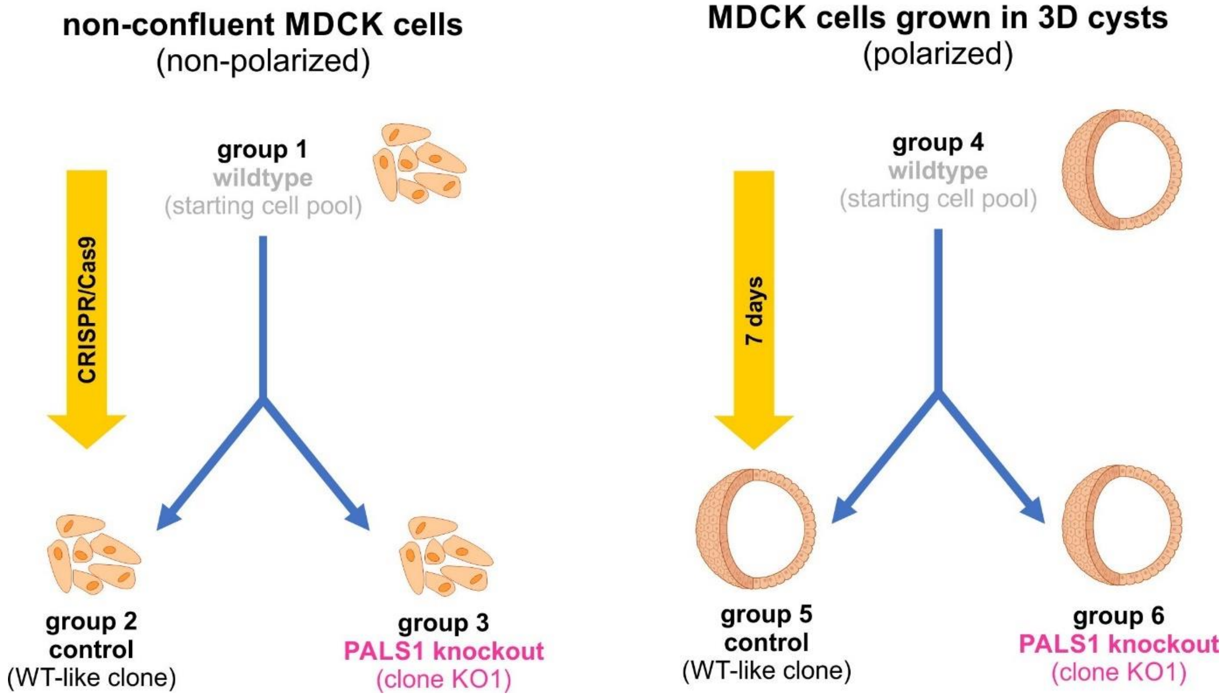

Fig. SF1: Preparation of mRNA samples for transcriptome analyses.

A: Scheme of used transcriptomes. Initially the analysis included six groups of transcriptomes samples: The first three groups (1-3) are samples from cells grown under non-confluent conditions (upper panel). *group 1:* transcriptomes derived from the starting pool of non-confluent MDCK II cells (wildtype non-confluent, n=4). *group 2*: Samples derived from a clone that ran through a CRISPR/Cas9 but maintained a wildtype *PALS1* gene (Ctr non-confluent, n=4). *group 3*: samples derived from a *PALS1* knockout clone (KO1 non-confluent, n=4) (Groh et al., 2024). The next are three groups (4-6) are samples derived from cells grown for 7 days in 3D cysts. *group 4*: 3D-cyst from the MDCK starting pool (WT-nc, n=4).*group 5*: Samples derived from 3D cysts of the clone that run through a CRISPR/Cas9 but maintained a wildtype PALS1 gene (Ctr-3D, n=3). *group 6*: Samples derived from 3D cysts of the KO1 clone (KO1 3D, n=3).

Supplemental Tables (titles and descriptions)

Table ST1 MDCK transcriptomes of wildtype, PALS1 knockout and the starting MDCK cell pool.
File name: ST01_S1_sample_description_030424.xlsx
Description of data: List of samples with sample ID from GEO and their description.

Table ST2A Differential expressed genes (DEGs) of subset A (WT-3D vs WT-nc)
File name: ST02A_sbst7_DESeq2_DEG_Ctr_3D_7d_vs_Ctr_2D_nconf_011223
Description of data: see name of table

Table ST2B DEGs of subset B (KO-3D vs KO-nc)
File name: ST02B_sbst7_DESeq2_DEG_KO_3D_7d_vs_KO_2D_nconf_011223
Description of data: see name of table

Table ST2C DEGs of subset C (KO-3D vs WT-3D)
File name: ST02C_sbst7_DESeq2_DEG_KO_3D_7d_vs_Ctr_3D_7d_011223
Description of data: see name of table

Table ST2D DEGs of subset D (KO-nc vs WT-nc)
File name: ST02D_sbst7_DESeq2_DEG_KO_2D_nconf_vs_Ctr_2D_nconf_140824
Description of data: see name of table

Table ST03 Shared DEGs of subset A and subset B
File name: ST03_Shared DEGs of Subset A and B
Description of data: Analyses of common and different DEGs of total, up-, and downregulated DEGs of subset A and B, including TOP 20 up- and downregulated DEGS.

Table ST04 Gene ontology biological processes (GO-BP) enrichment analysis for upregulated DEGs of subset A
File name:
ST04_UP_EnrichR_GO_Biological_Process_2021_sbst7_Ctr_3D_7d_vs_Ctr_2D_nconf_191223
Description of data: see name of table

Table ST05 GO-BP enrichment analysis for upregulated DEGs of subset B
File name:
ST05_UP_EnrichR_GO_Biological_Process_2021_sbst7_KO_3D_7d_vs_KO_2D_nconf_191223
Description of data: see name of table

Table ST06 REACTOME analysis for upregulated DEGs of subset A
File name: ST06_UP_EnrichR_Reactome_2016_sbst7_Ctr_3D_7d_vs_Ctr_2D_nconf_191223
Description of data: see name of table

Table ST07 REACTOME analysis for upregulated DEGs of subset B
File name: ST07_UP_EnrichR_Reactome_2016_sbst7_KO_3D_7d_vs_KO_2D_nconf_191223
Description of data: see name of table

Table ST08 KEGG analysis for upregulated DEGs of subset A
ST08_UP_EnrichR_KEGG_2021_Human_sbst7_Ctr_3D_7d_vs_Ctr_2D_nconf_191223
Description of data: see name of table
Table ST09 KEGG analysis for upregulated DEGs of subset B
ST09_UP_EnrichR_KEGG_2021_Human_sbst7_KO_3D_7d_vs_KO_2D_nconf_191223
Description of data: see name of table

Table ST10 GO-BP enrichment analysis for downregulated DEGS of subset A
File name:
ST10_DN_EnrichR_GO_Biological_Process_2021_sbst7_Ctr_3D_7d_vs_Ctr_2D_nconf_191223
Description of data: see name of table

Table ST11 GO-BP enrichment analysis for downregulated DEGs of subset B
File name:
ST11_DN_EnrichR_GO_Biological_Process_2021_sbst7_KO_3D_7d_vs_KO_2D_nconf_191223
Description of data: see name of table

Table ST12 REACTOME analysis for downregulated DEGs of subset A
File name: ST12_DN_EnrichR_Reactome_2016_sbst7_Ctr_3D_7d_vs_Ctr_2D_nconf_191223
Description of data: see name of table

Table ST13 REACTOME analysis for downregulated DEGs of subset B
File name: ST13_DN_EnrichR_Reactome_2016_sbst7_KO_3D_7d_vs_KO_2D_nconf_191223
Description of data: see name of table

Table ST14 KEGG analysis for downregulated DEGs of subset A
ST14_DN_Enrich_KEGG_2021_Human_sbst7_Ctr_3D_7d_vs_Ctr_nconf_191223
Description of data: see name of table

Table ST15 KEGG analysis for downregulated DEGs of subset B
File name: ST15_DN_EnrichR_KEGG_2021_Human_sbst7_KO_3D_7d_vs_KO_2D_nconf_191223
ST15_DN_EnrichR_KEGG_2021_Human_sbst7_KO_3D_7d_vs_KO_2D_nconf_191223
Description of data: see name of table

Table ST16 Shared DEGs of subset C and subset D
File name: ST16_Shared_DEGs_of_subset_C_and_D
Description of data: Analyses of common and different DEGs of total, up-, and downregulated DEGs of subset C and D, including TOP 20 up- and downregulated DEGS.

Table ST17 GO-BP enrichment analysis for upregulated DEGs of subset C
File name: ST17_UP_EnrichR_GO_Biological_Process_2021_sbst7_KO_3D_7d_vs_Ctr_3D_7d_191223
Description of data: see name of table

Table ST18 GO-BP enrichment analysis for downregulated DEGs of subset C
File name: ST18_DN_EnrichR_GO_Biological_Process_2021_sbst7_KO_3D_7d_vs_Ctr_3D_7d_191223
Description of data: see name of table

Table ST19 GO-BP enrichment analysis for upregulated DEGs of subset D
File name: ST19_UP_EnrichR_GO_Biological_Process_2021_sbst7_KO_2D_nconf_vs_Ctr_2D_nconf_140824.txt
Description of data: see name of table

Table ST20 GO-BP enrichment analysis for downregulated DEGs of subset D
File name: ST20_DN_EnrichR_GO_Biological_Process_2021_sbst7_KO_2D_nconf_vs_Ctr_2D_nconf_140824.txt
Description of data: see name of table

Table ST21 REACTOME analysis for upregulated DEGs of subset C
File name: ST21_UP_EnrichR_Reactome_2016_sbst7_KO_3D_7d_vs_Ctr_3D_7d_191223
Description of data: see name of table

Table ST22 REACTOME analysis for downregulated DEGs of subset C
File name: ST22_DN_EnrichR_Reactome_2016_sbst7_KO_3D_7d_vs_Ctr_3D_7d_191223
Description of data: see name of table

Table ST23 REACTOME analysis for upregulated DEGs of subset D
File name: ST23_UP_EnrichR_Reactome_2016_sbst7_KO_2D_nconf_vs_Ctr_2D_nconf_140824.txt
Description of data: see name of table

Table ST24 REACTOME analysis for downregulated DEGs of subset D
File name: ST24_DN_EnrichR_Reactome_2016_sbst7_KO_2D_nconf_vs_Ctr_2D_nconf_140824.txt
Description of data: see name of table

Table ST25 KEGG analysis for upregulated DEGs of subset C
File name: ST25_UP_EnrichR_KEGG_2021_Human_sbst7_KO_3D_7d_vs_Ctr_3D_7d_191223
Description of data: see name of table

Table ST26 KEGG analysis for upregulated DEGs of subset C
File name: ST26_DN_EnrichR_KEGG_2021_Human_sbst7_KO_3D_7d_vs_Ctr_3D_7d_191223
Description of data: see name of table

Table ST27 KEGG analysis for upregulated DEGs of subset D
File name: ST27_UP_EnrichR_KEGG_2021_Human_sbst7_KO_2D_nconf_vs_Ctr_2D_nconf_140824.txt
Description of data: see name of table

Table ST28 KEGG analysis for upregulated DEGs of subset D

File name:

ST27_UP_EnrichR_KEGG_2021_Human_sbst7_KO_2D_nconf_vs_Ctr_2D_nconf_140824.txt

Description of data: see name of table

Table ST29 Gene lists: cell junction, cell polarity, and cell-ECM interaction

File name:

ST27_UP_EnrichR_KEGG_2021_Human_sbst7_KO_2D_nconf_vs_Ctr_2D_nconf_140824.txt

Description of data: Basis for the three gene lists are gene included by known GO-terms.

For the gene list cell junctions, we used the following GO terms: GO:0043296 (apical junction complex, Cellular Component), GO:0070160 (tight junction, Cellular Component), and GO:0005912 (adherens junction, Cellular Component). The gene list of cell polarity genes based on the GO-term GO:0007163 (establishment or maintenance of cell polarity, Biological Process). For cell substrate interactions, cell adhesion or cell adhesion we used GO terms GO:0031589 (cell-substrate adhesion, Biological Process), GO:0007160 (cell-matrix adhesion, Biological Process) and the GO:0033627 (cell adhesion mediated by integrin, Biological Process).
